# Determination of dimensions of exfoliating materials in aqueous suspensions

**DOI:** 10.1016/j.mex.2015.12.002

**Published:** 2015-12-19

**Authors:** Anastasia L. Karpovich, Maria F. Vlasova, Natalya I. Sapronova, Valentin S. Sukharev, Victor V. Ivanov

**Affiliations:** Moscow Institute of Physics and Technology (State University), Institutskii per. 9, Dolgoprudny, Moscow Region 141700, Russia

**Keywords:** Determination of dimensions of exfoliating materials in aqueous suspensions, Exfoliation, Clays, Suspension, NMR relaxometry, Laponite, Size measurement

## Abstract

A method for measurement of dimensions of platy particles of exfoliating, or delaminating, materials, such as clays, in aqueous suspensions *in situ* is proposed. Equivalent spherical diameter (*esd*), measured by many common methods, depends more on the major (lateral) dimension of a particle, while it is less sensitive to changes of the particle thickness. Addition of the second method, results of which are a function of the particle diameter and thickness too, would provide more accurate determination of the particle dimensions. Previously, a combination of low-temperature nitrogen adsorption (BET) and dynamic light scattering (DLS) methods for determination of specific surface area of dry powder of platy particles and their *esd* in suspension was suggested. While such combination was suitable for measurement of particle size for non-exfoliating materials, it gave incorrect results for exfoliating materials, which dramatically change their surface area when dispersed in liquid. We modify this method by substituting BET method with NMR relaxometry, which allows to measure wetted surface area of the dispersed material directly in suspension. The advantages of this method are:

• More accurate determination of diameter and thickness of platy, particularly exfoliating, materials directly in suspension.
• Possibility of routine monitoring of particle size changes during the dispersing process.

## Graphical abstract

**Figure:**
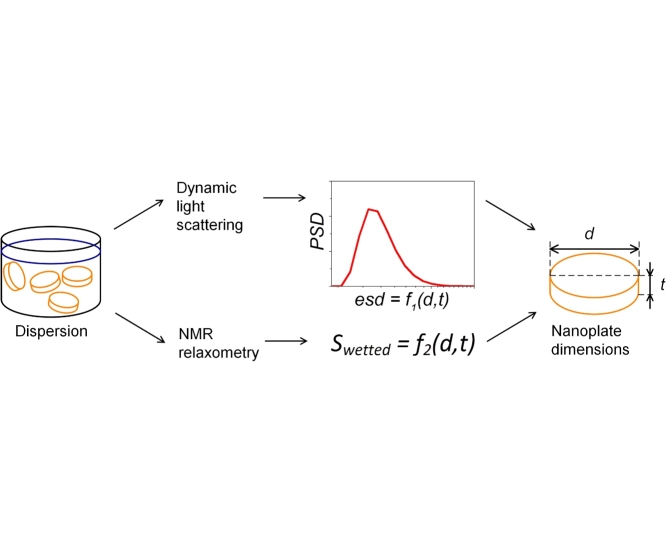

## Method

The method is a modification of previous work [1] for accurate determination of dimensions of particles of exfoliating materials dispersed in water and it is based on measurement of the specific surface area (*S*) of suspended particles by NMR relaxometry, and equivalent spherical diameter (*esd*) distribution of the particles by dynamic light scattering (DLS). Both measured values are functions of two particle dimensions *d* and *t* (lateral size and thickness). These dimensions can be then calculated from the system of two equations [1]:

(1) $$\left\{\begin{array}{c} d=\arctan\left(\frac{\text{d}}{t}\right)\cdot esd_{50}\cdot {\text{e}}^{-\ln^{2}\left(\frac{esd_{84}}{esd_{50}}\right)}\\ S=\frac{2}{\delta }\left(\frac{1}{t}+\frac{2}{d}\right) \end{array}\right)$$

where *δ* – density of the particles; *esd*_50_ is the particle size where 50% of the particles are finer than this size and *esd*_84_ is the particle size where 84% of the particles are finer than this size, which are routinely determined from *esd* distribution by DLS method.

The measured parameters in NMR relaxometry are time of spin-spin (*T*_2_) or spin-lattice (*T*_1_) relaxation of hydrogen protons. The specific surface area of a material dispersed in water depends on the relaxation time according to the following equation [2]:

(2) $$S=\left(\frac{1}{T_{i}}-\frac{1}{T_{fi}}\right)\frac{T_{bi}}{\varepsilon lC_{0}}$$

where *Ti* is the spin relaxation time of hydrogen protons in the dispersion, *T*_*fi*_ is the relaxation time of hydrogen protons in free (bulk) water, *T*_*bi*_ – spin relaxation time of hydrogen protons in water bound to the particle surface, *i* is 1 (spin-lattice) or 2 (spin-spin), *l* – thickness of the bound water layer, *ɛ* – fraction of the particle surface area covered with bound water, *C*_0_ – w/v concentration of the dispersed particles.

Measurement of *T*_*fi*_ and *T*_*i*_ is straightforward and requires simple measurement of the relaxation times of hydrogen in the particle-free water and the dispersion, respectively. At the same time the determination of the ratio *T*_*bi*_/*ɛl* is more elaborate, but fortunately needs to be found only once for a certain material. It can be determined in two ways:

1. Measurement of the relaxation time of hydrogen protons in water bound to the particle surface *T*_*bi*_ if the mechanism of water binding on the particle surface is known, and there is information about *l* and ɛ (direct measurement of which is out of scope of this paper). In general, *l* can be found from literature [3], for many clays *l* is about 1 nm [4]. If the particle surface is homogenous, *ɛ* = 1. If there are any active adsorption centres on the particle surface, ɛ can be estimated on the basis of amount and size of the adsorption centres.
2. If a sample of dispersion with completely exfoliated material is available (calibration sample), the ratio *T*_*bi*_/*ɛl* can be calculated from the equation:
  (3) $$\frac{T_{bi}}{\varepsilon l}=\frac{S_{C}\rho C_{C}}{\frac{1}{T_{Ci}}-\frac{1}{T_{fi}}}$$
  where *S*_*c*_ – specific surface area, calculated from AFM measurements of the particles from the dried calibration sample; *C*_*c*_ – w/v concentration of the particles in calibration sample; *T*_*ci*_ – relaxation time in the calibration sample.

As for the choice between measurements of spin-lattice (*T*_1_) or spin-spin (*T*_2_) relaxation time, *T*_2_ is usually more suitable for the less concentrated suspensions as its dependence on the specific surface area is more pronounced, while measurement of *T*_1_ may be required for measurements of relaxation in hydrogen spins in bound water, because *T*_2_ may be too small for such measurements [2]. It should be noted that spin-lattice and spin-spin relaxation time measurement should not be intermixed in one series of calculations.

As an example, we describe the detailed experimental procedure for this method for determination of geometrical dimensions of synthetic hectorite clay Laponite in aqueous dispersion. Laponite is a widely used model material for studies of exfoliating clays due to its high dispersibility in water, narrow size distribution and uniform shape of its individual disc-shaped platelets. Laponite powder consists of stacks, or tactoids, which exfoliate into platelets with diameter of about 20–25 nm and thickness of 1 nm, particle density *δ* = 2.53 g/cm^3^ [5], [6], [7].

The 0.5 w/v% Laponite dispersion was prepared by slowly adding 0.1 g of Laponite powder (Laponite RD, BYK Additives & Instruments) to 20 ml of deionized water with pH adjusted to 10 with 1 M NaOH. The dispersion was stirred for 45 min by magnetic mixer at speed of 1000 rpm. Then the dispersion was filtered through Whatman syringe filter (pore size of 0.1 μm) to remove large agglomerates. The measurements were performed for 1-day-old dispersion.

**Fig. 1:**
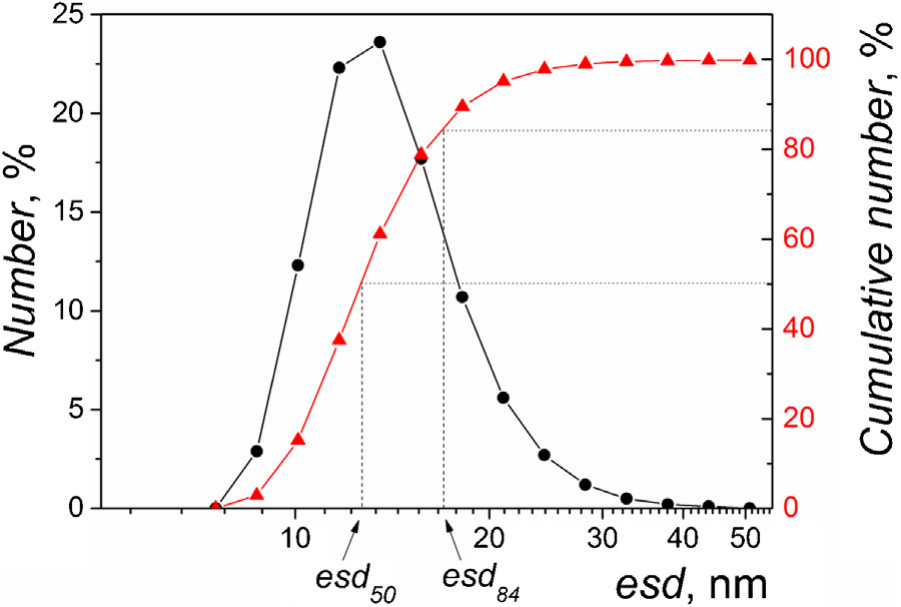
Particle *esd* distribution (black circles) and cumulative number (red triangles) particle *esd* distribution measured by dynamic light scattering for the Laponite suspension. (For interpretation of the references to color in this figure legend, the reader is referred to the web version of the article.)

DLS measurements were carried out on Zetasizer Nano ZS instrument (Malvern) at backscattering angle of 175° and the laser wavelength of 632.8 nm. The autocorrelation function was automatically processed by cumulant method. The particle size distribution obtained by DLS is shown in Fig. 1. Number particle size distribution was measured several times and for each distribution values *esd*_50_ and *esd*_84_ were calculated. Average value of *esd*_50_ was 14 nm and average value of *esd*_84_ was 18.4 nm.

^1^H NMR-relaxometery was performed on a portable bench-top device Acorn Area particle analyzer (Xigo Nanotools), operating at the resonant frequency of 13.0 MHz. Measured spin relaxation times were: *T*_*f*1_ = 2.82 s, *T*_*f*2_ = 2.63 s in Laponite-free water and *T*_1_ = 2.58 s, *T*_2_ = 1.09 s in Laponite dispersion.

The ratio *T*_*bi*_/*ɛl* was determined by two methods. The first approach is based on measuring the relaxation time of bound water *T*_*b*1_ using the powder with different amounts of adsorbed water and the second approach is based on calculation of *T*_*b*2_/*ɛl* using a calibration sample. In the first method we chose spin-lattice relaxation time (*T*_1_) measurements, because the values of spin-spin relaxation time (*T*_2_) were too small for the accurate measurements of *T*_*b*_. In the second method, we chose spin–spin relaxation time (*T*_2_) measurements as it is more sensitive to the changes of the surface area of the dispersed material, thus providing more precise results.

## Method 1

To measure spin–lattice relaxation *T*_1_ of the hydrogen spins in water molecules adsorbed on the surface of Laponite powder, the powder was degassed for 5 h at 350 °C. Then the dried powder was placed into a sealed container filled with moist air for varied periods of time to adsorb different amounts of water, and then poured into the NMR tube, which was placed into the NMR relaxometer, where *T*_1_ was measured. The amount of adsorbed water was determined by the mass difference between the wet and the degassed dry powders.

**Fig. 2:**
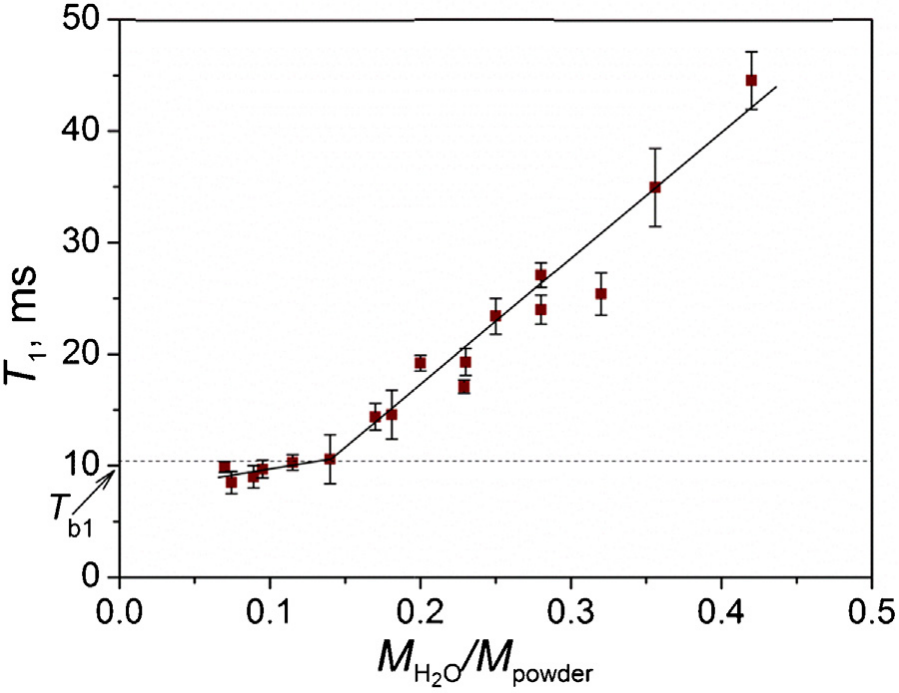
Dependence of spin–lattice relaxation time of hydrogen protons in powder with adsorbed water layer *T*_1_ on the ratio of mass of water $M_{{\text{H}}_{2}\text{O}}$ adsorbed on the powder to weight of the powder *M*_powder_. *T*_b1_ – Spin–lattice relaxation time of protons in bound water molecules. The lines serve as a guide to the eye.

Fig. 2 shows the dependence of *T*_1_ on the water weight fraction in wetted powder. According to the two-phase fast-exchange model, the spin relaxation time *T*_*i*_ corresponds to *T*_*fi*_ and *T*_*bi*_ by the following equation [2], [3]:

(4) $$\frac{1}{T_{i}}=\frac{1}{T_{fi}}\frac{V_{f}}{V}+\frac{1}{T_{bi}}\frac{V_{b}}{V}$$

where *V*_*f*_ – volume of bulk water, *V*_*b*_ – volume of bound water, *V* – total water volume.

Eq. (4) states that until all the water molecules belong to the surface bound water layer, *T*_1_ changes minimally, but starts to grow when the surface layer becomes saturated and adsorption of molecules not bound to the surface begins. Thus, the point of inflection of the plot on Fig. 2 corresponds to *T*_*b*1_ of 10.5 ms.

The thickness of the bound water layer *l* = 1 nm for Laponite particles in water [2], [3]. On the surface of Laponite platelet there are pronounced centres of binding of water molecules, sodium ions, which have the energy of binding significantly greater than the surface oxygen atoms have [2]. Surface concentration of Na ions is *α* = 0.7 nm^−2^ and radius of hydrated ion is *R*_Na_ = 0.21 nm [2]. Therefore, the fraction of the particle surface area covered with the bound water $\varepsilon =\alpha \pi R_{\text{Na}}^{2}=0.097$.

Considering *l* = 1 nm, *ɛ* = 0.097 and *T*_*b*1_ = 10.5 ms we get *T*_*b*1_/*ɛl* = 0.108 s/nm.

## Method 2

The second approach for calculating *T*_*b*2_/*ɛl* is to use to a calibration sample with known surface area. Laponite suspension (0.1 w/v%, 7 days old) was chosen for this purpose because particles completely exfoliate at this concentration [8].

The sample was diluted to 0.001% concentration and 10 μl of this diluted sample was deposited on a mica substrate (1 cm^2^) and dried at 150 °C. Before measurements the substrate was placed in a water vapor for 1 h to eliminate the charge influence and dried. Number average particle diameter measured by AFM in tapping mode (NTEGRA Aura scanning probe microscope, NT-MDT) was 19 nm, average thickness of a single particles was 1.05 nm.

Calculated from the AFM data, specific surface area *S*_*c*_ = 840 m^2^/g. Time of spin–spin relaxation of the calibration sample *T*_*c*2_ = 1.90 ms. Using these values in Eq. (3) *T*_*b*2_/*ɛl* is equal to 5.7 × 10^−3^ s/nm.

Substituting the obtained values of *T*_1_, *T*_*f*1_ and *T*_*b*1_/*ɛl* from the first method, or *T*_2_, *T*_*f*2_ and *T*_*b*2_/ɛ*l* from the second method to Eq. (2), we get the values of the specific surface area of Laponite particles of 710 and 620 m^2^/g, respectively, for the analyzed dispersion.

These values and *esd*_50_ and *esd*_84_ from DLS measurements were used in Eq. (1) to calculate diameter and thickness of Laponite particles dispersed in water. The results are presented in Table 1.

The difference of the calculated thickness value from the thickness of individual Laponite platelet (1 nm) indicates that the particles in the analyzed 1-day-old sample did not exfoliate completely, which is also demonstrated by smaller values of the specific surface area compared to the specific surface area of the calibration, completely exfoliated sample.

It should be noted that the thickness value from the second method is expected to be more accurate. The measurements and calculations of the bound water parameters in the first method introduce more uncertainty, associated with several assumptions of water binding mechanisms and determination of *T*_*b*1_ from the plot. If a calibration sample is available it is strongly recommended to use the second method.

In conclusion, we described a method suitable for simple determination of dimensions of various dispersed materials, especially of exfoliating, or delaminating materials, which change their surface area when dispersed, such as swelling clays, graphene, layered oxides, *etc.*, without their removal from the dispersion as in microscopy methods. The application of the method may be bounded by the need to understand the mechanism of the liquid binding to the particle surface, or the need for a calibration sample.

## Figures and Tables

**Table 1:** Results of determination of Laponite particles dimensions in aqueous dispersion by NMR relaxometry and DLS.

Method of determination *T*_*bi*_/ɛ*l*	Specific surface area, m^2^/g	Equivalent spherical diameter, nm		Diameter (*d*), nm	Thickness (*t*), nm
		*esd*_50_	*esd*_84_		
Method 1	710	14	18.3	20	1.1
Method 2	620	14	18.3	20	1.3
